# Efficacy of neonatal mouse muscle extracellular vesicles in skeletal muscle repair and regeneration

**DOI:** 10.1186/s13619-025-00274-6

**Published:** 2026-01-23

**Authors:** Chengwei Liu, Zhouyan Li, Xinyue Liu, Sitong Lv, Xijun Yin

**Affiliations:** Department of Animal Science, College of Agricultural, Yanbian University, Yanji, 133002 China

**Keywords:** Satellite cell, Neonatal mouse muscle extracellular vesicles, Muscle regeneration, Glycerol-induced injury, Extracellular vesicles-based therapy

## Abstract

Currently, effective treatments for skeletal muscle injury remain limited. The self-repair of skeletal muscle relies on the activation and differentiation of satellite cells (SCs), which fuse with damaged myofibers to form new fibers and thereby support muscle regeneration. However, in cases of severe injury, it is difficult for muscle tissue to fully restore its original structure and function, and its regenerative capacity is often markedly reduced. Thus, there is an urgent need to develop therapies that enhance muscle repair and restore physiological function. In this study, we investigated extracellular vesicles derived from neonatal mouse skeletal muscle (NMM-EVs), which are enriched in cargo from Pax7⁺ myogenic progenitor cells. We hypothesized that NMM-EVs could enhance SC activation and improve muscle regeneration following injury. Using glycerol-induced tibialis anterior (TA) muscle injury model, we evaluated the effects of intramuscular NMM-EV administration on skeletal muscle regeneration by histological, immunofluorescence, and functional analyses. In vivo, NMM-EVs significantly promoted skeletal muscle regeneration and functional recovery, upregulated Pax7 expression, increased the cross-sectional area and muscle mass of regenerated TA, and reduced fibrosis and fat infiltration. In vitro, NMM-EVs enhanced the proliferation and myogenic differentiation of mouse SCs and increased the expression of myogenic regulatory factors at both the mRNA and protein levels. In conclusion, this study demonstrates that NMM-EVs activate SCs within injured muscle, promote their proliferation and differentiation, and thereby accelerate injury repair and myofiber regeneration while attenuating fibrotic and adipogenic remodeling. These findings provide a scientific basis for the development of neonatal muscle–derived extracellular vesicle–based, cell-free therapeutic strategies for skeletal muscle injury.

**Supplementary Information:**

The online version contains supplementary material available at 10.1186/s13619-025-00274-6.

## Background

Skeletal muscle comprises 40%–60% of total body weight and is the largest organ in the human body. It plays a key role in locomotion, systemic metabolism, and energy homeostasis (Luo et al. 2024). As a highly plastic tissue, skeletal muscle possesses notable regenerative capacity following injury, primarily mediated by SCs (myoblasts situated between the sarcolemma and the basement membrane) (Morroni et al. 2023; Powers et al. 2020). These cells are activated under the regulation of myogenic factors (Pax7, MyoD, MyoG), undergo proliferation and differentiation into myoblasts, and ultimately fuse to form new muscle fibers. This process contributes to muscle growth as well as the remodeling and repair of injuries (Morroni et al. 2023). Given their central role, the regulation of SCs function has emerged as a promising therapeutic strategy for muscle repair (Duan et al. 2024).

However, severe muscle injury frequently results in pathological fibrosis and fat infiltration, where scar tissue and adipocytes replace functional muscle fibers. This condition reduces contractile strength and regenerative potential, leading to persistent weakness, diminished muscle strength, and reduced endurance. Therefore, developing effective strategies to facilitate muscle repair and regeneration remains a critical objective in biomedical research.

Recent advances in intercellular communication have revealed that extracellular vesicles (40–100 nm bilayer extracellular vesicles), are key mediators of cell-to-cell signaling. These vesicles transport proteins, RNA, and lipids to influence the behavior of recipient cells (Chiarello et al. 2018), and have demonstrated considerable potential in regenerative medicine. For example, extracellular vesicles derived from hematopoietic stem cells can facilitate the regeneration and functional recovery of damaged muscle fibers by delivering critical signaling molecules, confirming their therapeutic potential in muscle repair (Zeng et al. 2023). Extracellular vesicles secreted by skeletal muscle cells are enriched with myogenic regulatory factors (such as MyoD and Myogenin) and specific miRNAs (such as miR-1, miR-133, and miR-206), which can directly regulate the proliferation and differentiation of muscle precursor cells (Bittel and Jaiswal 2019; Mytidou et al. 2021). Further, extracellular vesicles derived from mesenchymal stem cells contain numerous anti-inflammatory factors, effectively attenuating muscle inflammation responses and providing a supportive microenvironment for tissue repair, thereby promoting muscle regeneration (Marote et al. 2016). Extracellular vesicles from differentiated skeletal muscle cells can also accelerate muscle regeneration and functional recovery by releasing bioactive molecules, including growth factors and miRNAs, which promote the proliferation and differentiation of muscle precursor cells (Wei et al. 2025).

Based on these findings, it was hypothesized that extracellular vesicles derived from muscle tissue may regulate muscle repair, improve muscle tissue quality and regeneration following injury. Satellite cells in injured muscles are normally activated as part of the endogenous regenerative response. Notably, studies have demonstrated that Pax7^+^ satellite cell activity is elevated in neonatal mice, and extracellular vesicles derived from neonatal muscle exhibit high bioactivity, allowing for further promoting satellite cell activation. Moreover, neonatal myogenic cells can revert to a Pax7^+^ stem cell-like state (idSCs) with enhanced regenerative potential (Price et al. 2025). This developmentally specific high expression of Pax7 suggests that neonatal muscle-derived extracellular vesicles are rich in pro-myogenic miRNAs (such as miR-206 and miR-1) and growth factors, which can not only effectively activate satellite cell proliferation and myogenic differentiation but also synergistically enhance muscle regeneration by inhibiting fibrotic pathways such as TGF-β and modulating the immune microenvironment including the upregulation of the anti-inflammatory factor IL-10 (Li et al. 2025; Mytidou et al. 2021; Wu et al. 2015; Zhang et al. 2025).

Therefore, we speculated that intramuscular injection of extracellular vesicles derived from neonatal mouse muscle could modulate the proliferation and differentiation of SCs thereby promoting muscle repair and regeneration. To investigate this, the present study employed a glycerol-induced muscle injury model, which is widely used to simulate fibrosis and adipogenesis. Glycerol disrupts sarcolemmal integrity, induces the infiltration of fibroadipogenic progenitor cells (FAPs), and impairs regeneration (FAPs), and impairs regeneration (Mahdy et al. 2018; Pisani et al. 2010). This study aimed to elucidate extracellular vesicles-mediated interactions among muscle tissue cells and evaluate the role of extracellular vesicles in muscle injury repair, with the goal of providing a novel therapeutic strategy for the treatment of muscle injury.

## Results

### Identification of extracellular vesicles from neonatal mouse muscle

**Fig. 1:**
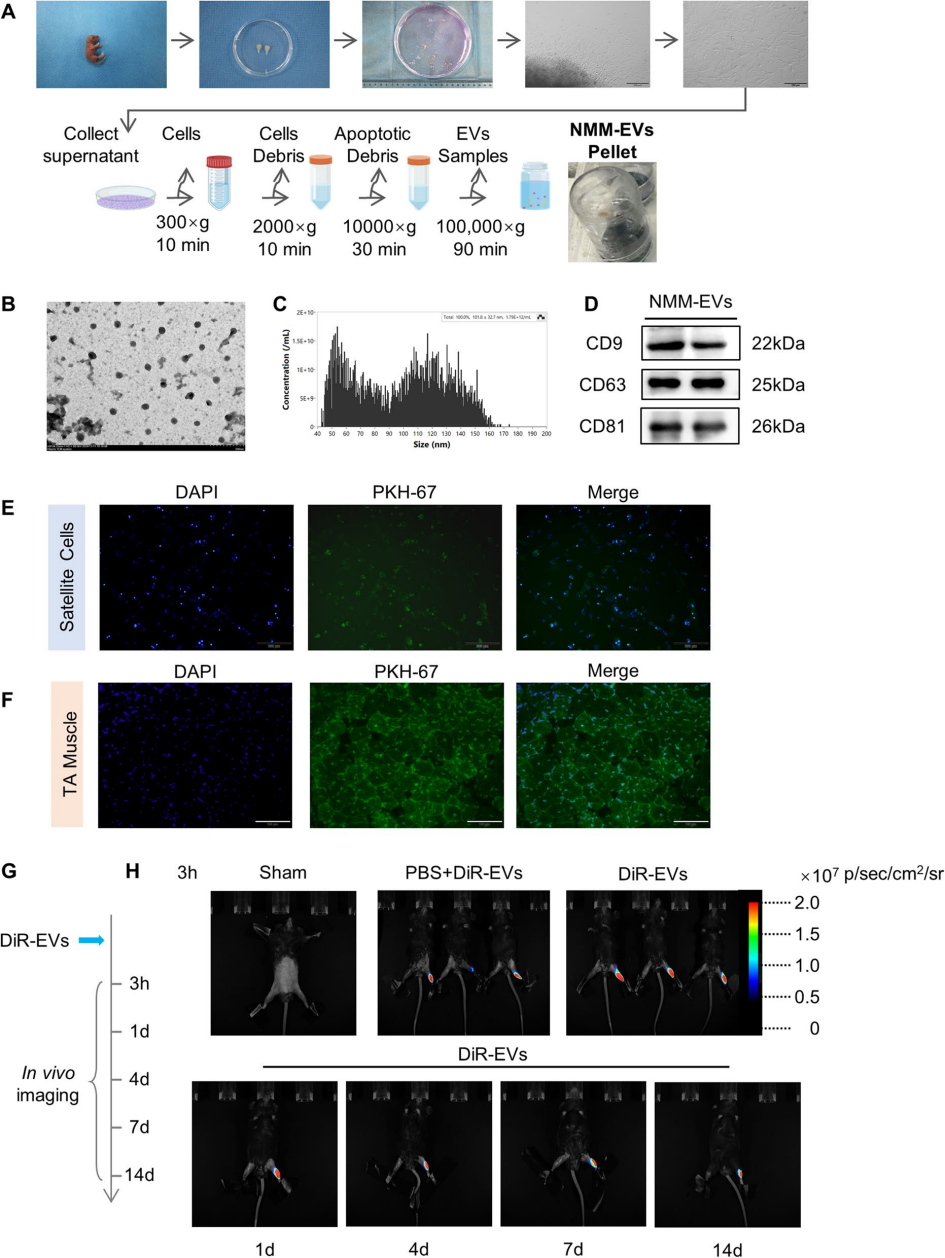
Production and identification of NMM-EVs. **A** Schematic diagram of the extracellular vesicles preparation process from neonatal muscle progenitor cells (MPCs). **B** Transmission electron microscopy images confirming the typical morphology of extracellular vesicles. **C** Nanoparticle tracking analysis showing particle size distribution. **D** Western blot detection of NMM-EVs surface markers CD9, CD63, and CD81. **E**, **F** Uptake of PKH67-labeled NMM-EVs (green) by satellite cells (Scale bar = 200 μm) and mouse tibialis anterior (TA) muscle (Scale bar = 100 μm). NMM-EVs are labeled with PKH-67 (green), DAPI was used to stain nuclei (blue). **G**, **H** NMM-EVs were labeled with 2 μM DiR, and residual free dye was removed. C57BL/6 mice received intramuscular injection of extracellular vesicles into the tibialis anterior muscle; dye-PBS and PBS. Representative IVIS images of the tibialis anterior muscle of mice at 3 h, 1 day, 4 days, 7 days and 14 days show their uptake

To investigate the role of extracellular vesicles in skeletal muscle regeneration following injury, neonatal mouse muscle-derived extracellular vesicles (NMM-EVs) were isolated and characterized (Fig. 1A). One-day-old neonatal mice were euthanized, and hind limb muscles were dissected and cultured. After 24 h, culture supernatants were collected, and NMM-EVs were isolated via differential ultracentrifugation. The morphology and characteristics of the extracellular vesicles were confirmed using TEM, NTA, and Western blotting. TEM analysis revealed the characteristic cup-shaped morphology of extracellular vesicles (Fig. 1B). NanoFCM showed a peak particle diameter between 50 and 120 nm (Fig. 1C). Western blot analysis confirmed the enrichment of canonical extracellular vesicle markers, including CD9, CD81, and CD63 (Fig. 1D). To assess biodistribution in vivo, PKH67-labeled extracellular vesicles were injected into the TA muscle. At 24 h post-injection, fluorescence microscopy revealed prominent accumulation of labeled extracellular vesicles in TA tissues (Fig. 1E). In parallel, PKH67-labeled extracellular vesicles were applied to cultured SCs. After 24 h of incubation, PKH67-labeled extracellular vesicles fluorescence signals were observed within cells, indicating effective cellular uptake (Fig. 1F). These findings confirm the successful isolation, characterization, and functional internalization of NMM-EVs both in vitro and in vivo (Fig. 1G, H). In addition, DiR, a near-infrared fluorescent lipophilic dye, was used to label NMM-EVs to monitor their retention in mouse muscle. Three hours after intramuscular injection, in vivo fluorescence imaging was observed on DiR-labeled NMM-EVs, as well as on DiR-labeled PBS and PBS alone (as controls). At 1, 4, 7, and 14 days after intramuscular injection, the DiR fluorescence signal in the TA muscle gradually decreased over time but remained detectable at day 14. These results indicate that NMM-EVs can persist within the injured muscle for at least 14 days after administration, providing experimental support for their sustained therapeutic effects on muscle regeneration.

### NMM-EVs enhance skeletal muscle histological recovery following muscle injury

**Fig. 2:**
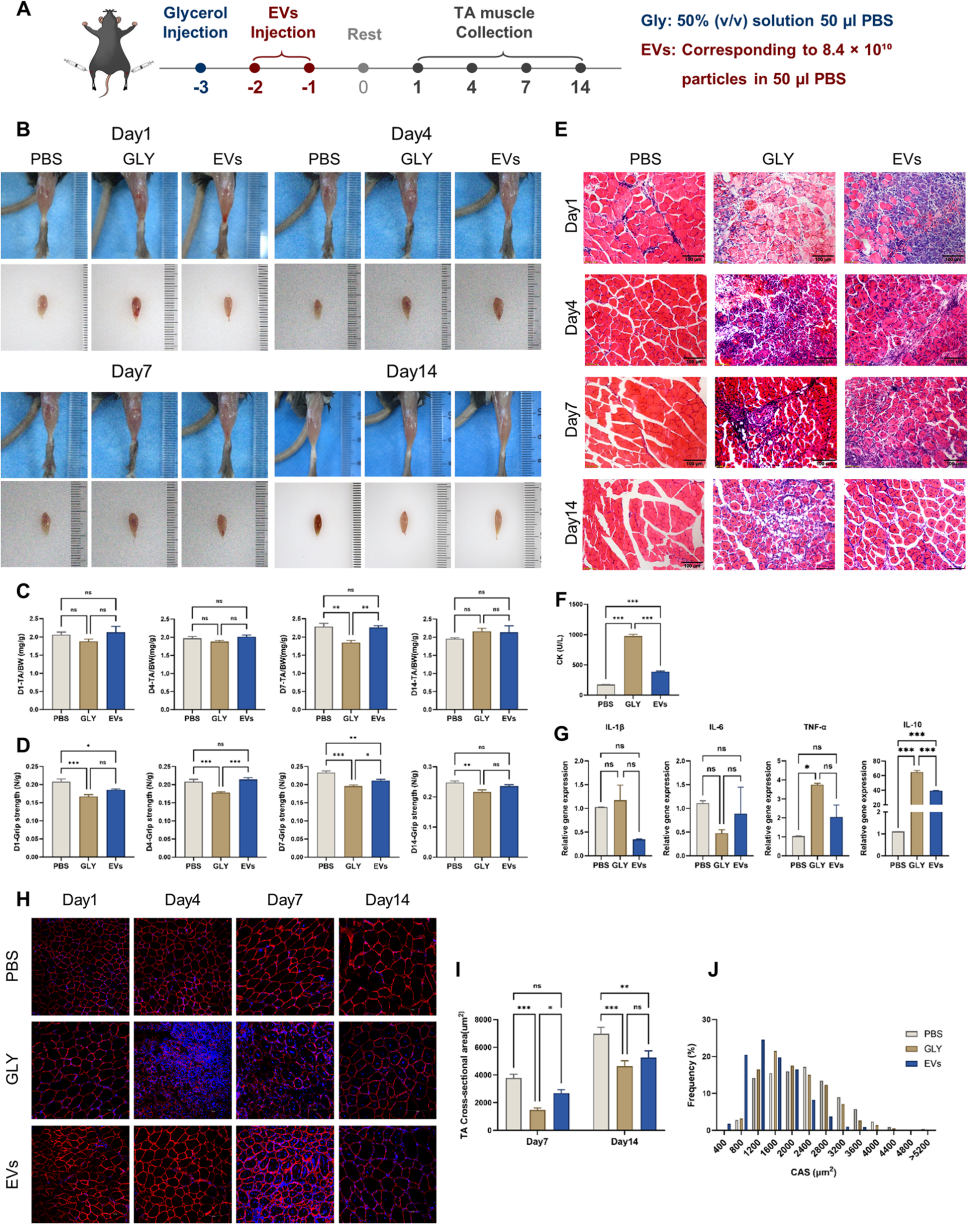
NMM-EVs promote repair of the tibialis anterior muscle after acute injury. Eight-week-old male wild-type C57BL/6 mice received two intramuscular injections of NMM-EVs following acute glycerol-induced muscle injury. **A** Schematic illustration of the muscle injury model treated with neonatal mouse muscle-derived extracellular vesicles. **B**–**D** Morphological changes, relative wet weight ratio (TA muscle weight/body weight), and grip strength of the TA muscle were assessed on days 1, 4, 7, and 14 post-injury. (*n* = 6, all compared with each group). **E** HE staining results after two treatments. In the PBS group, myocytes appeared tightly packed with nuclei located peripherally. In the injury group, newly formed myofibers exhibited centrally located nuclei, accompanied by prominent inflammatory cell infiltration. In contrast, the extracellular vesicles-treated group showed reduced infiltration and improved myofiber morphology. Scale bar = 100 μm. **F** Serum creatine kinase (CK) levels in the PBS, GLY (glycerol), and Exos groups (*n* = 6, all compared with each group). **G** Real-time PCR analysis of inflammatory and anti-inflammatory gene expression (TGF-β, IL-1β, IL-6, and IL-10) in uninjured muscle, immediately post-injury, and one day following extracellular vesicles treatment. (*n* = 4, all compared with each group) **H** Immunofluorescence staining of LAMININ (red) in TA muscle sections from PBS, GLY, and Exos groups (scale bar = 150 μm). **I** Quantification of the cross-sectional area of nucleated fibers at the center of each field of view. (*n* = 4, all compared with each group). **J** Histogram showing distribution of muscle fiber diameters in each group on day 7 in PBS, GLY, and Exos groups. All data are analyzed using one-way ANOVA and are expressed as mean ± SEM. *p < 0.05, **p < 0.01, ***p < 0.001, ns = not significant

To investigate the potential role of extracellular vesicles in muscle regeneration, we examined their effect on the regenerative response following glycerol-induced acute skeletal muscle injury. The model used to assess tibialis anterior (TA) injury was characterized by myofibril membrane disruption and permeability alterations induced by glycerol injection. The experimental timeline is illustrated below: Three days before the start of the evaluation period, glycerol (GLY) was injected into the tibialis anterior (TA) to induce acute injury. On the following 2 consecutive days, muscle-derived extracellular vesicles (EXO) or PBS were injected intramuscularly. TA muscle was collected on days 1, 4, 7, and 14 after injury for further analysis (Fig. 2A). Previous studies have shown that skeletal muscle exhibits a pronounced regenerative response within the first two weeks after injury, characterized by active myogenesis and tissue remodeling. Therefore, we selected days 1, 4, 7, and 14 after injury as key time points to evaluate the effects of NMM-EVs. Following two administrations of extracellular vesicles, the tibialis anterior (TA) in the glycerol-treated group showed significant congestion, swelling, and hematoma in the early stages (day 1 and day 4). In contrast, the extracellular vesicle treatment group showed reduced redness and swelling, improved muscle morphology, and approached that of undamaged tissue (Fig. 2B). On days 7, there were significant differences in the relative mass of the tibialis anterior (TA) muscle (calculated as TA muscle weight divided by body weight) between the groups (Fig. 2C). This indicates that extracellular vesicle treatment contributes to the recovery of muscle mass during muscle regeneration. To further assess functional recovery, we measured hindlimb grip strength. In the early stages of injury, grip strength decreased in both the glycerol and extracellular vesicle groups; however, the grip strength of mice in the extracellular vesicle treatment group was significantly enhanced compared to the glycerol group (Fig. 2D). By day 14, grip strength in the extracellular vesicle group had recovered to baseline levels, while that in the glycerol group had not fully recovered, indicating that extracellular vesicles can improve functional recovery during muscle repair. HE staining histological analysis (Fig. 2E) showed extensive myofiber damage in the injury group on day 1, including cellular debris in the interstitial spaces and significant inflammatory cell infiltration. By day 4, the myofibers in the glycerol group showed further degeneration, becoming smaller and rounder, with persistent inflammation and exhibited a high density of inflammatory cells (identified by their small, dark nuclei) within the interstitial space between muscle fibers, indicating ongoing inflammation. In contrast, the extracellular vesicle treatment group showed better preservation of muscle structure and reduced inflammatory cell infiltration. By day 7, inflammation in the extracellular vesicle group further subsided, and centrifuges were observed in many muscle fibers, indicating active regeneration. By day 14, centrifuges in both groups began to migrate to the periphery of muscle fibers, consistent with the maturation stage of muscle repair. Creatine kinase (CK), an intracellular enzyme released into the bloodstream after muscle injury, serves as a biomarker for muscle damage (Freitas et al. 2007; Wang et al. 2022). We measured serum creatine kinase levels in the three groups of mice on day 4. Compared to the group treated with glycerol alone, serum creatine kinase (CK) levels were significantly lower after extracellular vesicle treatment (Fig. 2F), indicating that extracellular vesicles help reduce inflammatory cell infiltration during the muscle injury repair process. Quantitative PCR analysis (Fig. 2G) showed that RNA extracted from the muscles of the three groups of mice on day 1 showed a significant increase in TNF-α mRNA expression through real-time quantitative PCR analysis. The increase in TNF-α mRNA expression was weakened in the extracellular vesicle treatment group, while the expression of IL-1βmRNA was slightly reduced, indicating that extracellular vesicles reduced the expression of these inflammatory cytokines. However, the mRNA expression levels of IL-6 and IL-10 were contrary to our expectations; we believe this result suggests that the anti-inflammatory effects of extracellular vesicles require further investigation over time (Nara and Watanabe 2021; Wang et al. 2014). Furthermore, analysis of muscle fiber cross-sectional area (CSA) on days 7 and 14 showed that the CSA values of mice in the extracellular vesicle treatment group were significantly increased compared to the glycerol group (Fig. 2H, I). Frequency distribution analysis of muscle fiber size showed a significant increase in the proportion of larger muscle fibers in the NMM-Exo treatment group (Fig. 2J). This finding supports the conclusion that NMM-Exo can promote muscle fiber regeneration and structural recovery. In summary, these results indicate that extracellular vesicles can improve muscle morphology, suppress inflammation, promote muscle fiber growth, and facilitate functional recovery in a glycerol-induced injury model.

### NMM-EVs promote skeletal muscle regeneration after injury by targeting Pax7 to activate muscle stem cells

**Fig. 3:**
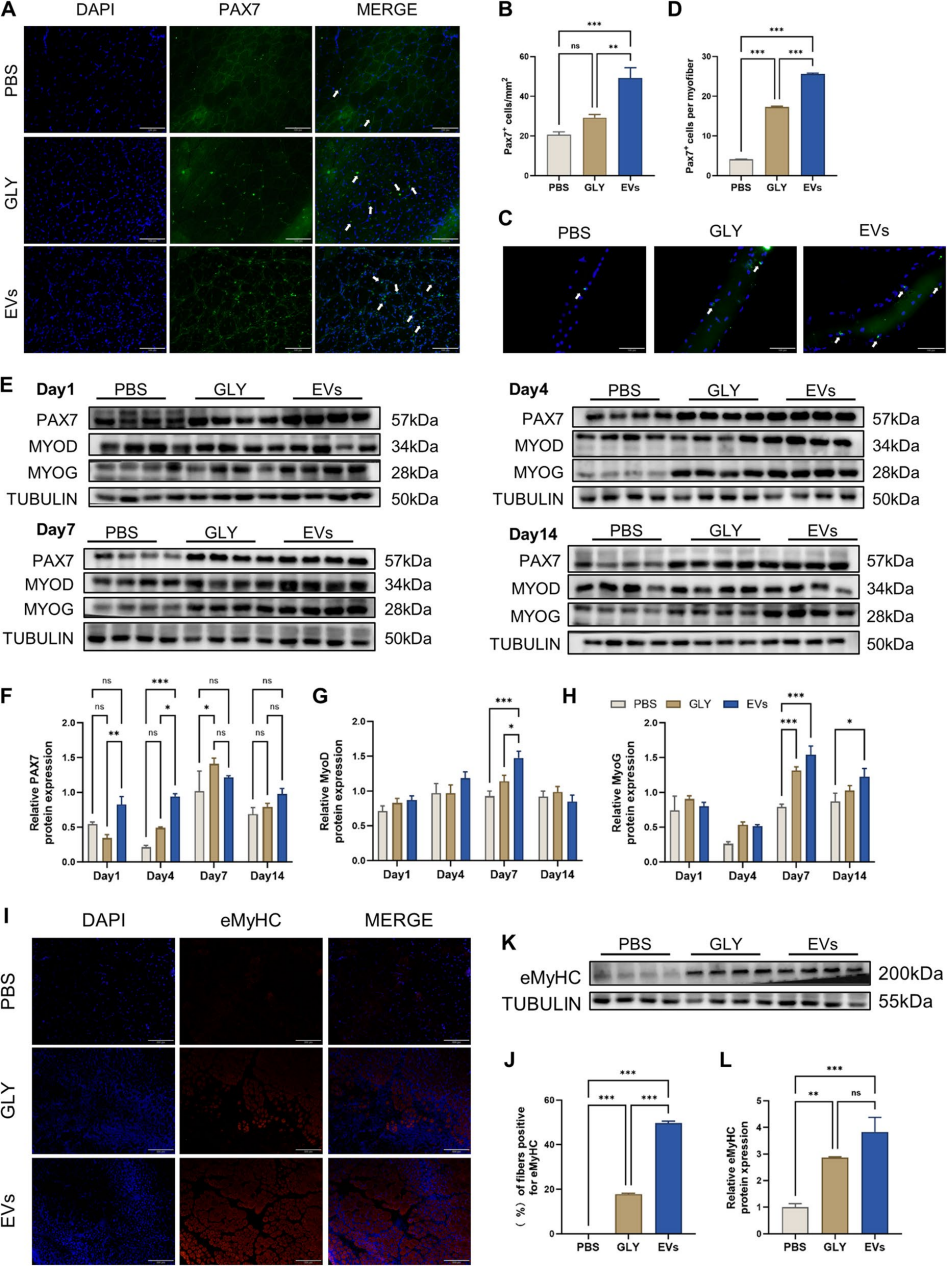
NMM-EVs activate muscle satellite cells to promote tibialis anterior muscle repair. **A**, **B** Immunofluorescence staining showing the localization and activation of satellite cells (SCs) post-injury. In the injury group, SCs become activated under stress signals and migrate from their niche to the interstitial space between damaged muscle fibers. (Scale bar = 100 μm, *n* = 6, all compared with each group). **C**, **D** Single myofiber isolation and immunofluorescence staining 7 days post-injury revealed a significant increase in Pax7⁺ SCs in the extracellular vesicles group compared to the injury-only group. (Scale bar = 100 μm, *n* = 6, all compared with each group). **E**–**H** Western blot analysis showing the expression of PAX7, MYOD, and MYOG during various post-injury stages. All densities of all protein bands were normalized to their corresponding Tubulin loading controls. Extracellular vesicles treatment significantly elevated the expression of these myogenic markers, particularly during early regeneration. (*n* = 4, all compared with each group). **I**, **J** Immunofluorescence staining of embryonic myosin heavy chain (eMyHC) illustrating increased numbers of regenerated myofibers in the extracellular vesicles-treated group (Scale bar = 200 μm). **K**, **L** Related eMyHC Western blot data supported these findings. All densities of all protein bands were normalized to their corresponding Tubulin loading controls. (*n* = 4, all compared with each group). All data are analyzed using one-way ANOVA and are expressed as mean ± SEM. *p < 0.05, **p < 0.01, ***p < 0.001, ns = not significant

We further investigated the effects of extracellular vesicles on SCs in injured skeletal muscle, considering their central role in muscle regeneration through activation, proliferation, and differentiation. Immunofluorescence staining for Pax7 (Fig. 3A, B) was performed on TA muscle samples collected at day4 to quantify self-renewing cells. Glycerol injection induces acute muscle injury, which in turn activates quiescent satellite cells to initiate muscle repair. On day 4 after glycerol-induced muscle injury, compared with the glycerol group, an increased number of Pax7-positive (Pax7^+^) cells were observed in the extracellular vesicle treatment group, with satellite cells being released from the quiescent sarcoplasmic reticulum, indicating that extracellular vesicles promote the activation and proliferation of satellite cells in the early stages of regeneration (Jia et al. 2024; Zeng et al. 2023).

In support of these findings, analysis of single muscle fibers isolated on day 4 post-injury and stained for Pax7 (Fig. 3C, D) showed a significantly higher number of Pax7⁺ cells in the extracellular vesicles-treated group than in the glycerol group, further confirming effective SC activation and transition into a proliferative state.

Western blot analysis of myogenic markers Pax7, MyoD, and MyoG was conducted at multiple time points (Fig. 3E, H) (Azhar et al. 2022; Lepper et al. 2011). On day 1, when inflammatory activity is highest, Pax7 and MyoD expression levels were significantly elevated in the extracellular vesicles group compared to the glycerol group, indicating early activation and proliferation of SCs. MyoG expression, typically low during initial phases, increased at later stages. On day 4, in extracellular vesicle-treated mice, the number of Pax7⁺ SCs increased significantly compared to the injury group, particularly during the early post-injury period, indicating that NMM-EVs transiently promote SC proliferation during the critical initial stages of regeneration. Day 7, for Pax7: compared to the glycerol group, its expression begins to decline during the differentiation of some satellite cells into myoblasts and their fusion to form myotubes. However, a portion of Pax7 + cells remains to replenish the satellite cell pool. For MyoD: as cells transition from proliferation to differentiation, MyoD levels remain high. For MyoG: Expression is strongly upregulated because MyoG is a key regulator of terminal differentiation and myotube formation. By day 14, differences between the groups diminished, and MyoG expression stabilized, reflecting the final stages of muscle fiber differentiation. However, the number of Pax7⁺ quiescent SCs remained elevated in the extracellular vesicles-treated group. This finding supports the hypothesis that NMM-EVs not only promote satellite cell proliferation but also enhance self-renewal, leading to an expanded SC reservoir for sustained regenerative capacity.

In parallel, eMyHC staining and Western blotting on day 7 showed that the positive area of newly formed regenerated myofibers in the extracellular vesicles group was significantly increased compared with the glycerol group, and the expression of eMyHC protein was significantly increased (Fig. 3I–L). This indicates that extracellular vesicles also promote SC differentiation into myoblasts and their subsequent fusion into regenerating myofibers. Day 7 represents a critical window during which myoblasts begin to fuse and mature into functional fibers. The upregulation of eMyHC during this phase reflects active myogenic differentiation and the rebuilding of contractile muscle tissue.

### NMM-EVs accelerate muscle regeneration and attenuate adipocyte infiltration and collagen deposition in vivo

**Fig. 4:**
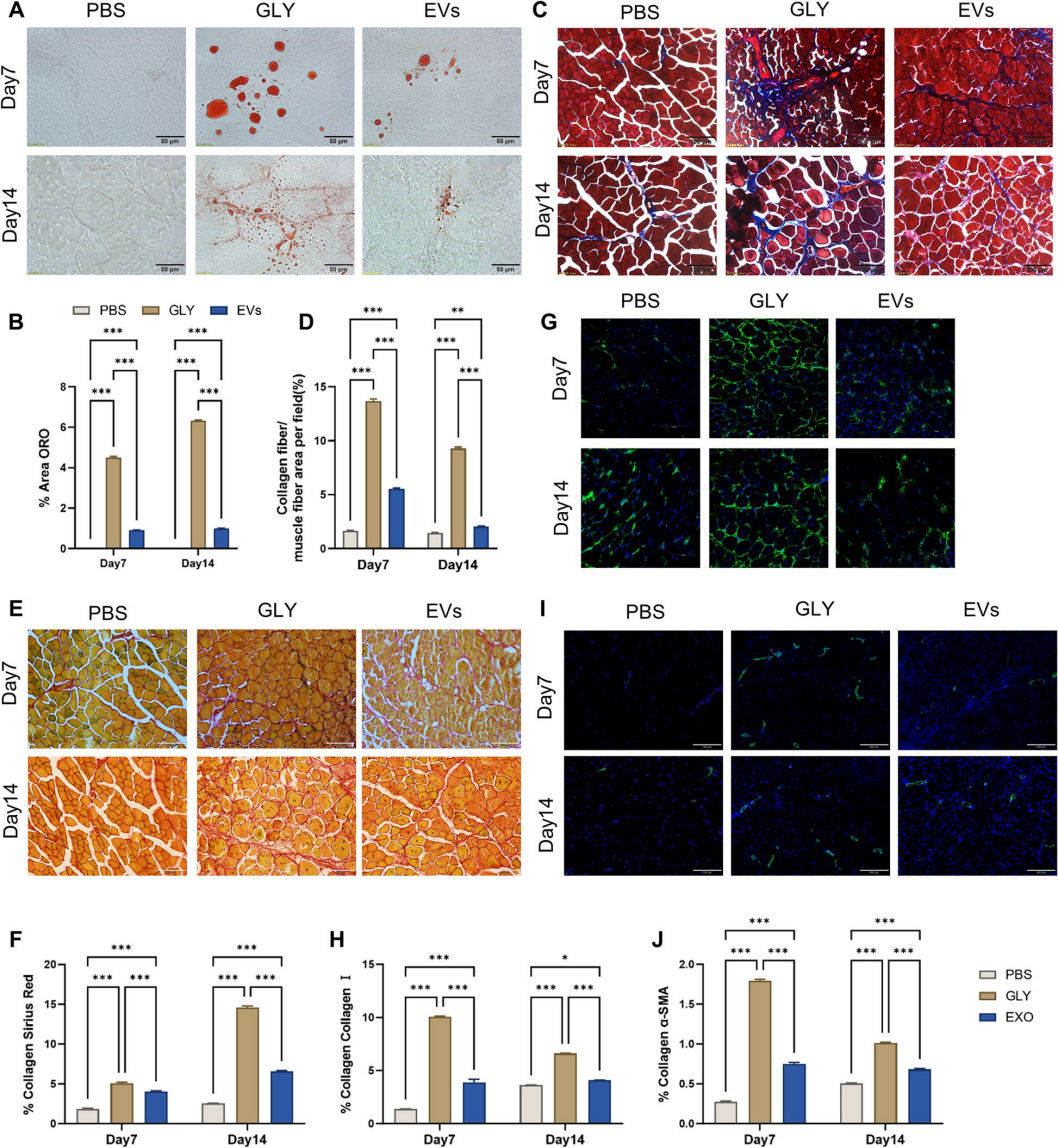
NMM-EVs accelerate muscle regeneration and reduce adipocyte infiltration and collagen deposition in vivo. **A**, **B** Quantification of adipocyte infiltration in tibialis anterior (TA) muscle tissue among the control (CON), glycerol-treated (GLY), and extracellular vesicles-treated (Exos) groups, based on Oil Red O staining (Scale bar = 50 μm, *n* = 6, all compared with each group). **C**–**F** Analysis of collagen fiber area in TA muscle sections stained with Masson's trichrome, Sirius red (*n* = 6, Scale bar = 100 µm). In Masson's trichrome-stained sections, muscle fibers are stained red, collagen fibers are stained blue, and nuclei are stained dark brown/black. In Sirius Red-stained sections, collagen fibers appear red/orange against a black background. **G**–**J** Immunofluorescence for Collagen I (Collagen I is shown in green, and nuclei are stained with DAPI (blue)) and α-SMA (α-SMA is shown in green, and nuclei are stained with DAPI (blue)) in PBS, GLY, and Exos groups (*n* = 6, Scale bar = 150 µm, 200 µm, all compared with each group). All data are analyzed using two-way ANOVA and are expressed as mean ± SEM. *p < 0.05, **p < 0.01, ***p < 0.001

Extracellular vesicles (EVs) reduce fat infiltration during muscle regeneration by modulating inflammation, activating satellite cells, inhibiting adipogenesis, and promoting myogenic differentiation. These coordinated effects limit ectopic adipocyte accumulation and support efficient muscle tissue repair. Oil Red O staining (Fig. 4A, B) was used to assess adipocyte infiltration in TA muscle tissues. On both days 7 and 14 post-injury, the extracellular vesicles-treated group exhibited a significantly lower degree of lipid droplet accumulation compared to the glycerol group, indicating a reduction in fat infiltration. Muscle injury is typically associated with an inflammatory response that contributes to pathological changes at the injury site, including adipocyte proliferation and fibrotic tissue formation. These secondary effects can impair muscle function and diminish the regenerative capacity of the tissue. To assess fibrosis, we performed Masson’s trichrome staining, Sirius red staining, and immunofluorescence analyses of fibrotic markers. The results demonstrated that NMM-EVs markedly reduced fibrosis in injured muscle by limiting fibroblast activity and suppressing collagen synthesis. Specifically, immunofluorescence staining for Collagen I and α-SMA, two markers of fibrotic remodeling, revealed that fibrotic deposition in the muscle interstitial space was significantly decreased in the extracellular vesicles-treated group compared to the glycerol group at both day 7 and day 14 (Fig. 4C–J). These findings suggest that NMM-EVs not only mitigate fat accumulation but also reduce excessive extracellular matrix deposition, thereby preserving muscle tissue architecture and supporting functional recovery.

### NMM-EVs promote satellite cell (SC) migration and proliferation

**Fig. 5:**
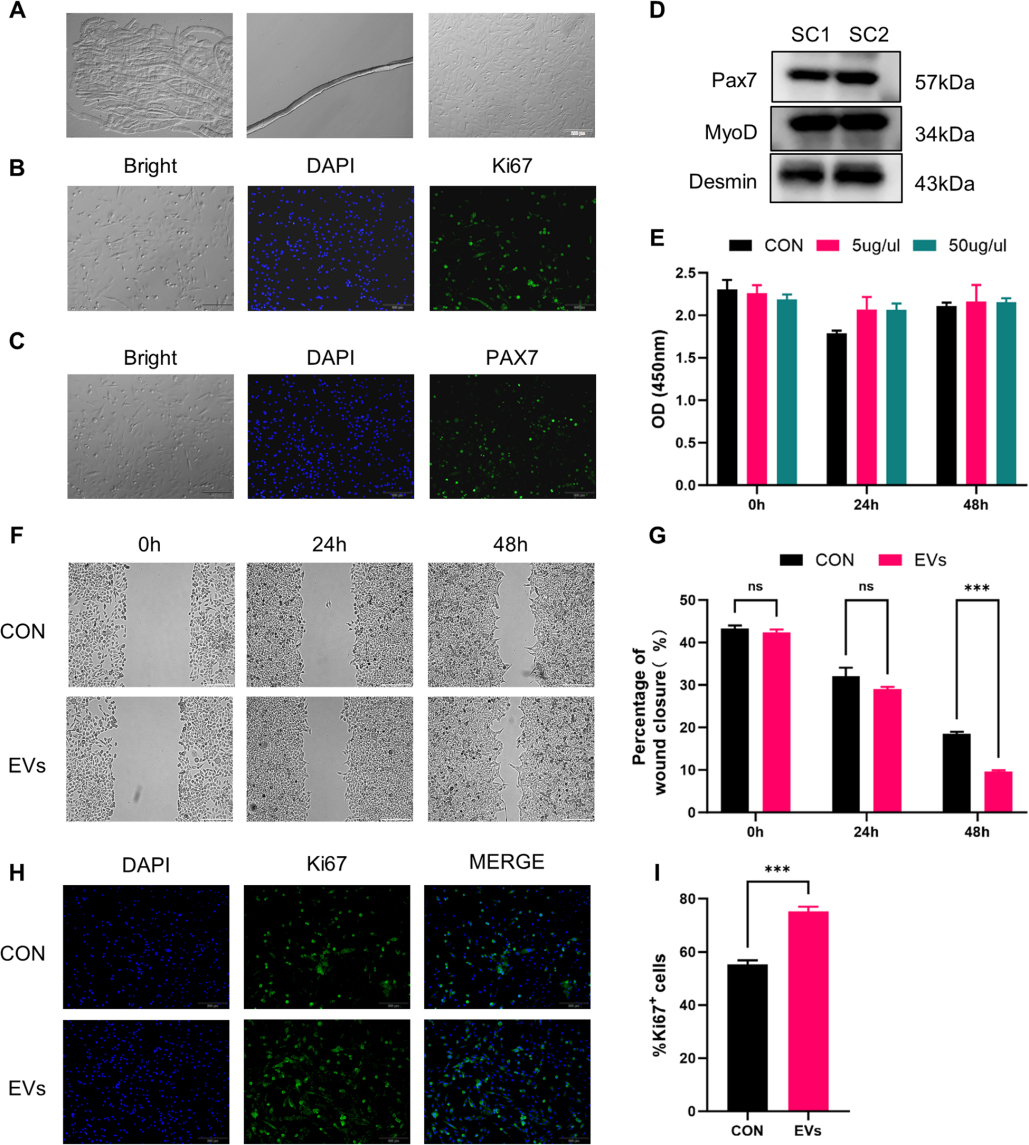
NMM-EVs promote the proliferation and migration of satellite cells. **A**–**D** Primary skeletal muscle satellite cells (SCs) were isolated from single myofibers of mouse hind limb muscle and cultured to the third generation. Cells were identified by immunofluorescence staining for Ki67 and Pax7, and the expression of satellite cell markers was confirmed by Western blotting (Scale bar = 100 μm). **E** CCK-8 assay results showing the proliferation of SCs treated with 5 μg/μl or 50 μg/μl of extracellular vesicles at 24 and 48 h (*n* = 6, all compared with each group). **F**, **G** Scratch wound assay demonstrating enhanced migration ability of SCs following extracellular vesicles treatment (Scale bar = 200 μm, *n* = 6, all compared with each group). **H**, **I** Immunofluorescence staining and quantification of Ki67^+^ cells at 48 h post-extracellular vesicles treatment, indicating increased proliferation (Scale bar = 200 μm, *n* = 6, all compared with each group). All data are analyzed using unpaired t-test and are expressed as mean ± SEM. *p < 0.05, **p < 0.01, ***p < 0.001, ns = not significant

To assess the effects of NMM-EVs on satellite cell function, we isolated single myofibers from the hind limbs of mice and extracted primary skeletal muscle SCs, which were expanded to the third generation for in vitro experiments (Fig. 5A). Immunofluorescence analysis revealed that over 87% of the isolated cells were PAX7⁺ and more than 83% were Ki67⁺ (Fig. 5B, C), confirming high purity and proliferative potential. Additionally, the cells expressed PAX7 and MYOD, further validating their identity as primary muscle SCs (Fig. 5D). Following treatment with extracellular vesicles, satellite cell proliferation increased in a dose-dependent manner. CCK-8 assays were performed at 24 and 48 h using two extracellular vesicle concentrations (5 μg/μl and 50 μg/μl). Both doses promoted proliferation; however, the difference between the two concentrations was not statistically significant (Fig. 5E). To evaluate migration, a scratch wound assay was conducted. Compared with the control group, the extracellular vesicles-treated group showed a significant enhancement in SC migration at both day 1 and day 2 post-treatment (Fig. 5F, G). Furthermore, immunofluorescence staining for Ki67 at 48 h post-extracellular vesicles exposure demonstrated a significantly higher proportion of Ki67⁺ cells in the extracellular vesicles group, suggesting that SCs entered the cell cycle more rapidly than those in the untreated control group (Fig. 5H, I). These findings indicate that NMM-EVs facilitate satellite cell proliferation and migration, contributing to a regenerative microenvironment that supports muscle repair (Cao et al. 2024).

### NMM-EVs promote myogenic differentiation of satellite cells

**Fig. 6:**
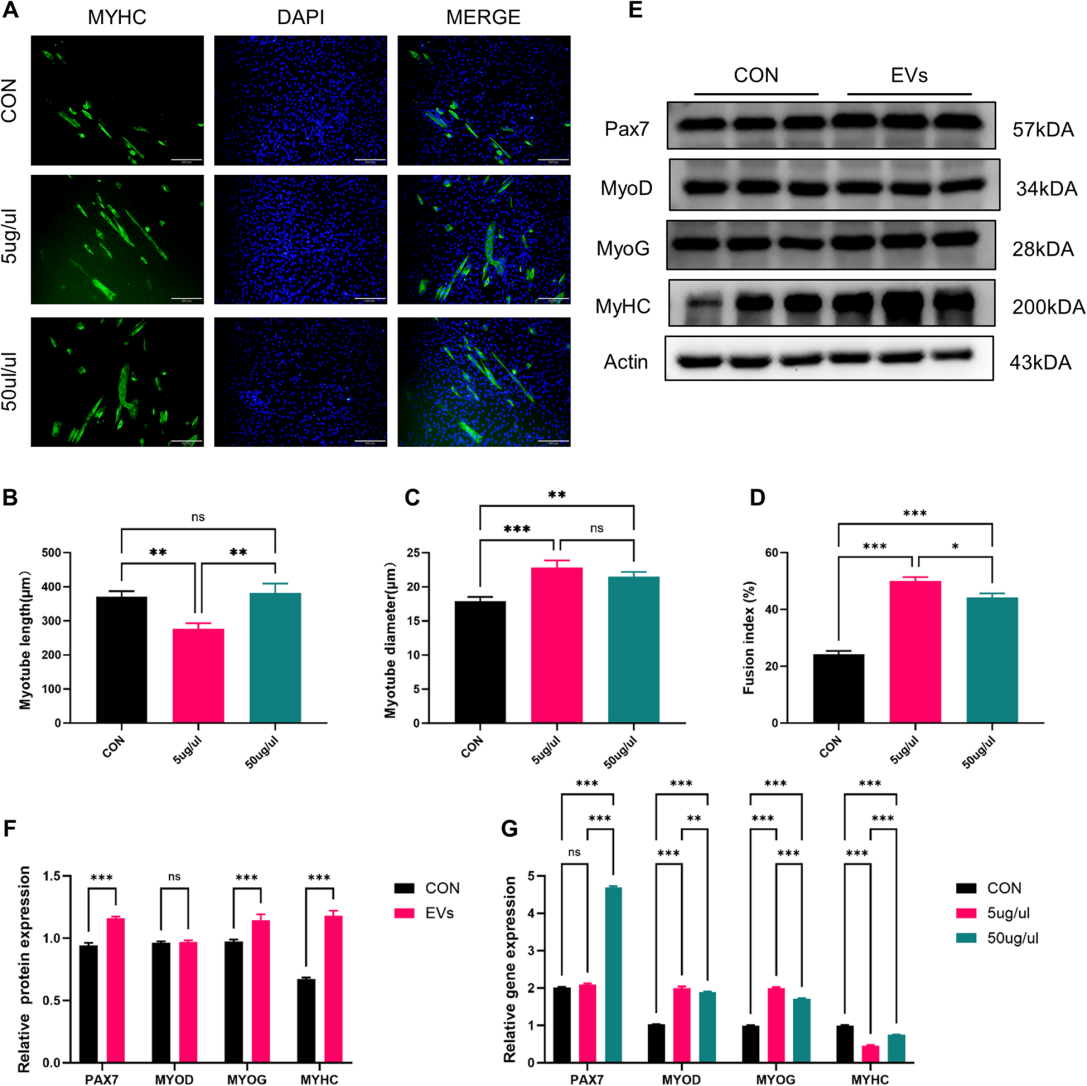
NMM-EVs can promote the differentiation and maturation of satellite cells. **A** Immunofluorescence staining of differentiated myoblasts for MYHC (green) following 5 days of extracellular vesicles treatment (Scale bar = 200 μm). **B**–**D** Quantitative analysis of myotube diameter, length and fusion index in satellite cells (SCs) treated with PBS, 5 μg/μL, and 50 μg/μL extracellular vesicles (Scale bar = 200 μm, *n* = 6, all compared with each group). **E**, **F** Western blot analysis and densitometric quantification of MYOD, MYOG and MYHC expression in muscle SCs from each treatment group. β-actin was used as a loading control (*n* = 4, all compared with each group). **G** Gene expression levels of MYOD, MYOG, and MYHC as determined by RT-PCR analysis of extracellular vesicles-treated SCs (*n* = 4, all compared with each group). All data are analyzed using one-way ANOVA, two-way ANOVA and are expressed as mean ± SEM. *p < 0.05, **p < 0.01, ***p < 0.001, ns = not significant

We have previously demonstrated that extracellular vesicles derived from neonatal mouse muscle can enhance the differentiation capacity of C2C12 in vivo (Liu et al. 2025), thereby contributing to the restoration of muscle function in models of muscle degenerative diseases such as sarcopenia. To determine whether a similar effect occurs in vitro, we treated differentiated satellite cells with two concentrations of extracellular vesicles (5 μg/μl and 50 μg/μl) for five days. Two concentrations were used here because we wanted to verify whether high concentrations of extracellular vesicles could promote better differentiation of myotubes from satellite cells (Hanson et al. 2023). We used myosin heavy chain (MYHC) immunofluorescence staining to assess myoblast differentiation. MYHC is a marker of terminal muscle differentiation, and its expression reflects myotube formation and maturation, thus serving as a reliable indicator of in vitro myoblast differentiation. The results showed that, compared with the PBS control group, the myotube diameter was significantly increased in the extracellular vesicle-treated group, while the myotube length did not differ significantly. Furthermore, the myotube fusion index was increased in the extracellular vesicle-treated group (Fig. 6A–D), indicating enhanced myotube formation (Fig. 6E, F) (Choi et al. 2016; Ono and Sakamoto 2017). Western blot analysis showed that, compared with the PBS control group, key myogenic markers (including MYOD, MYOG, and MYHC) were significantly upregulated in SCs treated with extracellular vesicles. These markers reflect different stages of myogenic differentiation and maturation, confirming that extracellular vesicle treatment enhances the differentiation process at the protein level. (Fig. 6G) Furthermore, qPCR analysis showed that, compared with the PBS group, the mRNA expression levels of MYOD, MYOG, and MYHC were significantly increased in the extracellular vesicle treatment group. These transcriptomic changes further support the conclusion that NMM-EVs enhances the differentiation capacity of SCs in vitro, consistent with and validating our previous in vivo results.

### NMM-EVs are enriched with myomiRs and promote muscle regeneration by targeting satellite cells

**Fig. 7:**
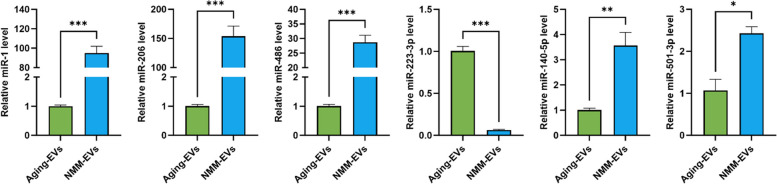
Comparative analysis of myomiR expression profiles in aging muscle-derived (Aging-EVs) and neonatal mouse muscle-derived (NMM-EVs) extracellular vesicles. Bar graph representing fold-change in miRNA expression in NMM-EVs relative to Aging-EVs. Data are presented as mean ± SEM (*n* ≥ 3 independent experiments). *p < 0.05, **p < 0.01, ***p < 0.001 vs. Aging-EVs (Student’s t-test)

Based on previous studies implicating specific muscle-enriched microRNAs (myomiRs) in Pax7 regulation, satellite cell function, and muscle regeneration, we selected six candidate myomiRs—miR-1, miR-206, miR-486, miR-223-3p, miR-140-5p, and mmu-miR-501-3p—for further analysis (Table 1)(Cao et al. 2024; Chen et al. 2010; Cheng et al. 2020; Dey et al. 2011; Mizbani et al. 2016). Total RNA was then extracted from extracellular vesicles derived from aged skeletal muscle (Aging-EVs) and neonatal mouse muscle (NMM-EVs). Compared with Aging-EVs, NMM-EVs exhibited significantly higher levels of miR-1, miR-206, miR-486, miR-140-5p, and mmu-miR-501-3p, whereas miR-223-3p was markedly reduced in NMM-EVs (Fig. 7). These results indicate that, relative to aging muscle–derived EVs, NMM-EVs are selectively enriched in a subset of pro-myogenic myomiRs that are involved in suppressing Pax7 translation, promoting satellite cell activation, and driving satellite cells toward the myogenic lineage.

**Table 1:** Key myomiRs involved in Pax7 regulation and skeletal muscle regeneration

miRNA	Mechanism Targeting Pax7	Effect on Muscle Regeneration	Reference
miR-1/miR-206	Directly binds to Pax7 3'UTR to inhibit translation, promoting satellite cell differentiation	Limits proliferative potential, enhances myogenic differentiation	Chen et al. 2010
miR-486	Modulates PI3K/AKT/mTOR pathway, affecting muscle satellite cell proliferation and differentiatio	Promotes myogenesis, inhibits muscle atrophy	Dey et al. 2011
miR-223-3p	Regulates inflammatory response, indirectly influencing Pax7^+^ satellite cell microenvironment	May inhibit muscle regeneration, associated with inflammatory myopathies	Cheng et al. 2020
miR-140-5p	Affects skeletal muscle differentiation-related pathways	Inhibits myogenic differentiation, potentially maintains stem cell quiescence	Cao et al. 2024
miR-501-3p	Specifically induced and highly expressed in activated myogenic progenitors (MPs) during muscle regeneration	Regulating myosin heavy chain (MHC) expression (specifically MYH3) during muscle regeneration	Mizbani et al. 2016

## Discussion

Skeletal muscle repair is a complex biological process involving coordinated phases of degeneration, inflammation, regeneration, and fibrosis. While skeletal muscle exhibits a strong regenerative ability after mild injuries, such as those related to physical activity, severe injuries often result in extensive fibrosis, fatty infiltration, and impaired functional recovery. A variety of in vitro and in vivo approaches have been investigated to enhance muscle regeneration by leveraging cellular and biochemical cues associated with this process (Järvinen et al. 2005; Longo et al. 2012).

The muscle healing process is classically divided into three overlapping phases: destruction, repair, and remodeling (Mulbauer and Matthew 2019). Following injury, necrosis of myofibers and infiltration of inflammatory cells occur. Subsequently, SCs are activated and proliferate to initiate muscle regeneration. These activated SCs differentiate into myoblasts, which then align and fuse to form multinucleated myotubes. These new myotubes may either fuse with existing myofibers that survived the injury or contribute to the formation of new fibers, which gradually mature and restore normal morphology and function (Relaix and Zammit 2012).

Day 1: Early Inflammation and Necrosis: This initial time point is crucial for assessing the acute injury response. Damaged muscle fibers undergo necrosis, releasing intracellular substances and triggering a rapid inflammatory cascade. Neutrophils initiate an immune response, followed by the recruitment of macrophages to the site of injury (Tidball 2005). This phase is marked by elevated levels of pro-inflammatory cytokines (e.g., TNF-α, IL-1β, IL-6) and increased serum creatine kinase (CK) activity, indicating impaired sarcoplasmic integrity and muscle damage (Peake et al. 2017). Furthermore, satellite cells receive inflammatory signals and are gradually released from the sarcoplasmic reticulum, initiating activation and the regeneration process. Therefore, we assessed CK levels and pro-inflammatory cytokine expression in the early stages to capture the peak of the initial injury response.

Day 4: Peak of Inflammation and Transition to Regeneration: On day 4 post-injury, the inflammatory response typically reaches its peak, characterized by the dominance of M1 macrophages (Arnold et al. 2007). Simultaneously, early satellite cell activation and proliferation can be detected, indicating the onset of muscle regeneration (Relaix and Zammit 2012).

Day 7: Myogenesis and Myofiber Regeneration: Day 7 represents the mid-stage of muscle regeneration, when satellite cells are activated, proliferate, and subsequently differentiate (Chargé and Rudnicki 2004). Simultaneously, the expression of key myogenic regulatory factors (MRFs) such as MyoD and MyoG in developing cells also reflects the status of muscle regeneration. Therefore, we assessed the TA cross-sectional area at this time to quantify the size of newly regenerated muscle fibers.

By day 14, the regeneration process transitions to the late stage of muscle remodeling and maturation. In regenerating muscle fibers, the centrally located nucleus gradually migrates to the periphery, indicating that it has completed terminal differentiation and structural organization (Wasgewatte Wijesinghe et al. 2019). Depending on the degree and severity of the initial injury, collagen deposition and fibrosis, as well as adipocyte infiltration, may occur at this stage, potentially affecting long-term muscle function and hindering the recovery of the muscle regeneration process. Therefore, we assessed the degree of muscle fibrosis in the TA at this time and the degree of fat infiltration (Mahdy et al. 2018; McHale et al. 2012).

Therapeutic strategies have included the use of myoblasts, SCs, and stem cells derived from various sources, often in combination with growth factors, to improve muscle regeneration and functional outcomes. For example, human adipose-derived stem cells cultured on gelatin-based scaffolds have demonstrated the ability to differentiate into myoblasts and support muscle regeneration in injury models. In the mouse soleus muscle loss model, the combination of muscle-derived stem cells with muscle growth factors significantly enhanced muscle recovery.

Given that myoblasts, SCs, and multipotent stem cells are capable of facilitating muscle repair, a key question is whether targeting the most primitive regenerative components could further improve outcomes. Mouse fetuses at a gestational age of less than 12 days represent a critical period of skeletal muscle development. During this stage, embryonic cells are rapidly proliferating and differentiating into various tissues, including muscle (Tichy et al. 2018). Muscle stem cells, such as myoblasts and SCs, are particularly active at this time, producing high levels of growth factors and cytokines essential for tissue regeneration and repair (Pawlikowski et al. 2017).

In our study, we confirmed by immunofluorescence staining and Western blot that SC expression in neonatal mouse muscle (3 days post-birth) was significantly higher than in aged mouse muscle. Furthermore, we demonstrated that intramuscular injection of NMM-EVs could activate quiescent SCs and enhance their expression (see Fig. S1). Neonatal muscle tissue during this period is rich in signaling molecules, such as fibroblast growth factor (FGF) and insulin-like growth factor (IGF), which promote cell proliferation, differentiation, and survival. In addition, transforming growth factor-β (TGF-β) and interleukins serve as important regulators of inflammation and repair (Shin et al. 2023; Wan et al. 2024). It is increasingly recognized that the regenerative effects of mesenchymal stem cells (MSCs) are largely mediated by their secreted factors, especially those carried in extracellular vesicles, rather than by the cells themselves. Accordingly, NMM-EVs contain numerous bioactive molecules, including miRNAs, proteins, and other signaling molecules that may enhance muscle regeneration (Henze et al. 2020). Extracellular vesicles derived from early-stage tissue sources may stimulate stem cell activation and differentiation. The growth factors they carry can promote satellite cell activation and proliferation, accelerating muscle fiber regeneration. Additionally, these extracellular vesicles help modulate the inflammatory microenvironment, reduce proinflammatory cytokine release, inhibit fibrosis, and thereby promote efficient tissue repair.

This study employed a glycerol-induced chronic muscle injury model to investigate the reparative effects of NMM-EVs on skeletal muscle. NMM-EVs were administered via local intramuscular injection on the first day post-injury, and outcomes were assessed over a 14-day period. We observed that SCs were rapidly activated within 24 h following glycerol-induced injury, coinciding with an acute inflammatory response in the injured tissue and early satellite cell proliferation. A substantial infiltration of inflammatory cells was also noted. By day 4, satellite cell proliferation had increased markedly, with cells beginning to migrate toward the injury site. At this stage, pronounced myofiber necrosis and inflammatory infiltration were observed, though these were notably reduced in the extracellular vesicles-treated group. By day 7, muscle necrosis had progressed, and satellite cell differentiation reached a peak. A large number of myoblasts accumulated at the injury site, initiating the reconstruction of new muscle fibers. Centrally nucleated myotubes, which are indicative of regenerating muscle, were more numerous in the extracellular vesicles group than in the untreated glycerol group, reflecting enhanced myogenic activity and improved tissue morphology. By day 14, the muscle entered the repair and remodeling phase. Satellite cell activity gradually declined, and the number of newly formed myofibers stabilized. In the extracellular vesicles-treated group, muscle structure was better preserved, and reductions in both fibrosis and adipocyte infiltration were evident. Overall, muscle regeneration appeared most pronounced in this group. In vitro experiments further supported these findings. NMM-EVs significantly enhanced the proliferation and migration of primary mouse SCs, promoted their differentiation, and increased the myotube fusion index, thus providing clear evidence of myogenic differentiation potential. Taken together, these findings indicate that NMM-EVs effectively promote muscle repair and regeneration in mice following injury.

SCs are essential for muscle regeneration throughout life. Upon injury, quiescent SCs residing beneath the basal lamina are activated, proliferate, and differentiate into myoblasts to support tissue repair. Numerous studies have elucidated the mechanisms by which SCs contribute to muscle regeneration. Beyond forming new myofibers, SCs modulate the local inflammatory environment through cytokine secretion, including IL-6 and TNF-α, mediating cross-talk with macrophages to facilitate the regeneration process (Yin et al. 2013). Further, both mechanical and biochemical cues following injury influence satellite cell dynamics, driving tissue remodeling and repair by regulating proliferation and differentiation (Li et al. 2009). These microenvironmental changes are critical for initiating the regenerative cascade and underscore the pivotal role of SCs in orchestrating skeletal muscle healing.

Under normal physiological conditions, SCs remain in a quiescent state. Upon muscle injury, they are activated, re-enter the cell cycle, and initiate myogenic programs leading to proliferation. A subset of these cells differentiates into myocytes to support tissue repair. This process involves the synchronous activation and co-expression of Pax7 and MyoD. Most proliferating SCs subsequently downregulate Pax7 and progress toward differentiation. However, a portion of these cells retains Pax7 expression while downregulating MyoD, exiting the differentiation pathway and returning to a quiescent state. This mechanism ensures the preservation of a satellite cell pool capable of supporting future regenerative needs (Wan et al. 2024). Given the critical role of SCs and their energy demands during regeneration, modulating cellular energy metabolism may offer a novel approach to enhance muscle repair.

During muscle regeneration, pathological outcomes, such as fibrosis and fat infiltration, frequently impair functional recovery and muscle quality. Inflammatory cells and cytokines are integral to the early phases of regeneration, contributing to the clearance of necrotic myofibers. However, persistent inflammation is detrimental and may inhibit effective regeneration. Key cytokines, such as IL-1β, IL-6, TNF-α, and IL-10, have been implicated in regulating myogenesis. IL-1β promotes myotube catabolism by activating nuclear factor κB (NF-κB) signaling and increasing the expression of atrophy-related proteins, such as atrogin-1/MAFbx and MuRF1 (O'Brien et al. 2020). Similarly, elevated TNF-α concentrations inhibit myogenic progression, while chronic IL-6 elevation has been linked to skeletal muscle atrophy. In contrast, IL-10, a major anti-inflammatory cytokine, suppresses pro-inflammatory mediators, such as IL-1β and TNF-α, thereby contributing to tissue homeostasis. In this study, glycerol injection led to the upregulation of pro-inflammatory cytokines (IL-1β, IL-6, and TNF-α) and downregulation of IL-10 during the early stage of muscle injury. These imbalances were observed at both early (day 7) and late (day 14) time points, suggesting that sustained inflammation may be a key factor contributing to suboptimal muscle regeneration.

Fibroblasts, under physiological conditions, synthesize extracellular matrix (ECM) components, such as collagen, to support structural integrity during tissue repair. However, excessive fibroblast activation or ECM deposition following injury can result in pathological fibrosis, reducing muscle elasticity and impairing contractile function (Dewi et al. 2025). Moreover, inflammatory cytokines such as TNF-α and IL-6 can also induce adipogenic differentiation, contributing to fat infiltration within injured muscle. In pathological states, severe muscle cell loss may lead remaining SCs to adopt adipogenic fates, increasing intramuscular fat deposition as a compensatory mechanism, especially in chronic or degenerative conditions (Tosic et al. 2018).

An unexpected observation during the study was that, when glycerol was injected into only one hindlimb, the contralateral, non-injected leg often exhibited signs of injury as well. This phenomenon may be attributable to systemic physiological responses and the influence of blood circulation. Glycerol, upon intramuscular injection, rapidly enters the bloodstream and is distributed systemically. Elevated circulating glycerol levels can alter systemic physiology by disrupting electrolyte balance and osmotic pressure, adversely affecting cellular homeostasis in distant tissues. In addition, inflammatory cytokines and damage-associated molecular mediators released at the injury site may disseminate via the circulatory system and affect uninjured muscles, leading to indirect damage. To mitigate these systemic effects and ensure experimental consistency, glycerol injections were administered bilaterally to both hindlimbs. This approach standardized injury severity across subjects and minimized confounding systemic influences, thereby enhancing the reliability of the experimental results.

One limitation of this study is that the specific molecular mechanisms by which extracellular vesicles influence satellite cell behavior remain unclear. Further research is required to elucidate the precise signaling pathways through which extracellular vesicles regulate satellite cell proliferation, differentiation, and contribution to muscle tissue regeneration. Another limitation lies in the in vitro model: unlike in vivo experiments, glycerol cannot be used to induce muscle damage in cultured cells. Therefore, in vitro experiments were limited to assessing whether extracellular vesicles could modulate satellite cell behavior in the absence of injury. Developing a reliable in vitro model that simulates muscle damage would enable direct comparison between in vivo and in vitro systems, providing more robust validation of the regenerative effects of extracellular vesicles. Further methodological advancements are needed to address this gap.

In summary, NMM-EVs were shown to promote regeneration and repair of glycerol-induced skeletal muscle injury in mice. These extracellular vesicles reduced inflammatory cell infiltration and attenuated fibrosis and fat accumulation in the damaged muscle. The regenerative effects were primarily mediated through the activation and expansion of SCs, which are essential for forming new myofibers following injury. Satellite cell activation is tightly regulated by a complex interplay of molecular signals and cellular interactions within their microenvironment, or niche. This niche encompasses not only the surrounding extracellular matrix but also a network of secreted factors and cellular signals that create a dynamic and responsive environment (Howard et al. 2020; Tu and Li 2023). Effective activation and contribution of SCs to muscle regeneration depend on the maintenance of niche homeostasis. This homeostasis is regulated by interactions among various cell types, including myocytes, fibroblasts, macrophages, and the signaling molecules, such as cytokines and extracellular vesicles, that they secrete (Dort et al. 2019). These interactions coordinate the processes of satellite cell proliferation, migration, and differentiation, which are essential for efficient muscle repair.

Moreover, sustaining the balance of the satellite cell niche is critical not only for facilitating timely muscle regeneration but also for preventing pathological outcomes such as chronic fibrosis or adipocyte infiltration. A deeper understanding of the signaling mechanisms underlying satellite cell activation and their ecological interactions within the niche holds significant potential for the development of targeted therapeutic strategies to enhance muscle regeneration and treat muscle-related disorders.

## Conclusions

This study demonstrated that extracellular vesicles derived from neonatal mouse muscle significantly enhance skeletal muscle regeneration in a glycerol-induced injury model by promoting the proliferation and differentiation of SCs. In addition, extracellular vesicle treatment was shown to reduce adipocyte infiltration and fibrosis, thereby supporting the formation and fusion of myotubes into regenerating muscle fibers. These findings provide a promising foundation for developing extracellular vesicle-based therapies aimed at enhancing muscle repair and treating musculoskeletal injuries.

## Materials and methods

### Animals

All animal procedures were approved by the Ethics Committee of Yanbian University (approval No. YD20240828013) and were conducted in accordance with the ARRIVE guidelines and international regulations for the care and use of laboratory animals, including the U.K. Animals (Scientific Procedures) Act 1986, EU Directive 2010/63/EU, and the National Research Council’s Guide for the Care and Use of Laboratory Animals. Eight-week-old male C57BL/6 mice were used in this study.

Humane endpoints were predefined as follows: (1) loss of more than 20% of initial body weight; (2) severe motor dysfunction, such as inability to obtain food or water independently; and (3) signs of open wound infection or necrosis. Animals that reached any of these endpoints were immediately euthanized by cervical dislocation or by an overdose of anesthetic agents, in accordance with institutional guidelines.

Seventy-two 8-week-old healthy male C57BL/6 mice were obtained from the Experimental Animal Center of Yanbian University and were housed under specific pathogen-free (SPF) conditions (24 ± 1 °C, 70% ± 5% humidity, 12 h light/dark cycle). All animals were acclimated for one week before experiments with ad libitum access to standard chow and water.

### Isolation of extracellular vesicles from neonatal mouse muscle

Extracellular vesicles (EVs) were isolated from neonatal mouse skeletal muscle as previously described (Kargl et al. 2023; Watanabe et al. 2022) with minor modifications. Neonatal C57BL/6 mice within 24 h of birth were humanely euthanized by rapid decapitation using sharp surgical scissors, followed immediately by cervical dislocation to ensure death. Immediately after euthanasia, carcasses were placed on ice at 4 °C for 5 min to preserve tissue. Under sterile conditions in a biosafety cabinet, hindlimb muscles were dissected, minced into approximately 1 mm^3^ pieces, washed with phosphate-buffered saline (PBS), and transferred to culture dishes. Serum-free Dulbecco’s modified Eagle’s medium (DMEM) was added, and the tissue explants were incubated for 24 h. Conditioned medium was then sequentially centrifuged at 300 × g, 2,000 × g, and 10,000 × g to remove cells and debris, and EVs were pelleted from the clarified supernatant by ultracentrifugation at 100,000 × g for 90 min. EVs were characterized by transmission electron microscopy (TEM) for morphology, nanoflow cytometry (NanoFCM) for size distribution and particle concentration, and Western blotting for EV surface markers (CD9, CD63, and CD81).

#### Transmission electron microscopy (TEM)

The morphology of EVs was examined using transmission electron microscopy. Briefly, 5 µL of purified EVs (approximately 100 µg/mL) were adsorbed onto a 200-mesh, glow-discharge-treated carbon-coated copper grid (Electron Microscopy Sciences, Hatfield, PA, USA) for 30 min. The grid was then fixed at room temperature for 1 h in a mixture of 2% paraformaldehyde and 2.5% glutaraldehyde (both from Electron Microscopy Sciences) in 0.1 M sodium dihydrogen phosphate buffer (pH 7.4). After fixation, the grid was washed three times with Milli-Q ultrapure water and negatively stained with 1% uranyl acetate (Electron Microscopy Sciences). The staining solution was freshly filtered through a 0.22 µm filter, and the grid was stained for 30 s. Excess stain was removed by gently blotting with Whatman filter paper, and the grid was allowed to air-dry completely in a desiccator. Images were acquired using a JEOL JEM-140 transmission electron microscope.

#### NanoFCM

For NanoFCM analysis, NMM-EV samples were diluted in particle-free PBS to a final concentration between 1 × 10⁷ and 1 × 10⁹ particles/mL, based on preliminary measurements. Prior to sample analysis, the NanoFCM Pro instrument (NanoFCM Ltd., Nottingham, UK) was calibrated using 68 nm silica microspheres provided by the manufacturer, following the manufacturer’s instructions. After calibration, the diluted EV samples were loaded onto the instrument. Instrument settings were as follows: laser power 30%, detector gain 600 V, and trigger voltage 50 mV. Data for each sample were acquired for 5 min, and each experimental condition was measured in triplicate. The acquired data were analyzed using NanoFCM Professional software (NanoFCM Ltd.), and EV size distribution and particle concentration were calculated based on the measured scattering intensity.

### Skeletal muscle injury

Skeletal muscle injury was induced in the tibialis anterior (TA) muscle using a glycerol injection model as previously described (Pisani et al. 2010). Briefly, glycerol was diluted in phosphate-buffered saline (PBS) to a final concentration of 50% (v/v). Mice were anesthetized with an intraperitoneal injection of sodium pentobarbital (50 mg/kg body weight) to achieve a surgical depth of anesthesia, confirmed by the absence of a pain reflex. To induce injury, 50 µL of 50% glycerol was slowly injected into the mid-belly of the TA muscle of both hindlimbs using an insulin syringe. This protocol reliably induces acute myofiber damage and subsequent regeneration. Animals were euthanized at 1-, 4-, 7-, and 14-days post-injury for tissue collection and subsequent analyses.

### Extracellular vesicles treatment

Mice were randomly assigned to three groups: (1) a control group, which received intramuscular injections of PBS without glycerol-induced injury; (2) a glycerol group, in which TA muscle injury was induced by glycerol injection and mice subsequently received PBS; and (3) a glycerol + EV group, in which TA muscle injury was induced by glycerol injection followed by treatment with neonatal mouse muscle–derived EVs (NMM-EVs).

The injury and treatment schedule are illustrated in Fig. 2A. Briefly, glycerol-induced TA muscle injury was performed 3 days before the start of the evaluation period. NMM-EV treatment was then administered on the following 2 consecutive days. For each injection, 50 µL of NMM-EVs was injected into each TA muscle, corresponding to a dose of 250 µg NMM-EVs per leg (8.4 × 10^1^⁰ particles in 50 µL PBS). Thus, each leg received two injections of NMM-EVs, 24 h apart. Day 0 was defined as 1 day after the second EV injection, and TA muscles were collected at 1, 4, 7, and 14 days after day 0 for tissue analysis.

### Isolation, culture, and identification of primary satellite cells

Three-day-old (postnatal day 3) C57BL/6 mice were euthanized by rapid decapitation in accordance with the ARRIVE guidelines, followed by brief immersion in 75% ethanol (≤ 30 s) for surface sterilization. Under sterile conditions in a biosafety cabinet, skeletal muscles (quadriceps, gastrocnemius, and tibialis anterior) were isolated from the hindlimbs. The tissues were finely minced with ophthalmic scissors and subjected to a two-step enzymatic digestion with 0.1% collagenase type I followed by 0.25% trypsin at 37 °C for a total of 30 min to obtain a single-cell suspension. After filtration through a 70 µm cell strainer, the suspension was centrifuged at 300 × g for 10 min, and the cell pellet was resuspended in Dulbecco’s modified Eagle’s medium (DMEM) supplemented with 20% fetal bovine serum (Gibco) and 1% penicillin–streptomycin.

Satellite cells were enriched by differential adhesion. Briefly, cells were plated for 30 min, and non-adherent cells were collected as the first fraction (P1); the pre-plating step was repeated to obtain P2–P3 fractions, which typically yield cultures with > 90% Pax7⁺ cells, as reported previously (Kim et al. 2020). Cells were maintained at 37 °C in a humidified incubator with 5% CO₂, and the medium was changed every 72 h. All procedures adhered to the 3R principles (replacement, reduction, and refinement) to promote animal welfare.

### Extracellular vesicles labeling with PKH67

#### In vitro uptake of NMM-EVs in SCs

Primary muscle satellite cells (SCs) were cultured to approximately 70%–80% confluence and incubated with PKH67-labeled NMM-EVs (50 µg EV protein per well) for 24 h. To prepare PKH67-labeled NMM-EVs, EVs were incubated with PKH67 dye in diluent buffer for 2 min at room temperature in the dark, after which the staining reaction was quenched with bovine serum albumin (BSA). Labeled EVs were purified by ultracentrifugation at 100,000 × g for 90 min and resuspended in PBS. After incubation with PKH67-labeled NMM-EVs, cell nuclei were counterstained with Hoechst 33,342, and EV uptake was evaluated by fluorescence microscopy based on the intracellular PKH67 signal.

#### In vivo uptake of NMM-EXO in Tibialis anterior muscle

To assess in vivo uptake of NMM-EVs, PKH67-labeled NMM-EVs (100 µg EV protein in 50 µL PBS) were injected into the tibialis anterior (TA) muscle of C57BL/6 mice. After 24 h, TA muscles were harvested, embedded in optimal cutting temperature (OCT) compound, snap-frozen in liquid nitrogen, and cryosectioned at 8 µm thickness. Sections were permeabilized and blocked with 1% bovine serum albumin (BSA) in PBS containing 0.1% Triton X-100, followed by nuclear counterstaining with DAPI. Fluorescence microscopy was used to visualize PKH67 fluorescence within muscle fibers.

#### DiR markers for NMM-extracellular vesicles

DiR labeling of NMM-EVs for in vivo tracking was performed as previously described (Mentkowski and Lang 2019). with minor modifications. DiR was dissolved in anhydrous ethanol to prepare a 2 mM stock solution, which was stored at − 20 °C protected from light. NMM-EVs were mixed with the DiR stock solution to a final dye concentration of 5 µM, gently vortexed, and incubated for 30 min at room temperature in the dark. Unbound dye was removed by ultracentrifugation at 100,000 × g for 90 min at 4 °C, and the EV pellet was washed once with 1 mL sterile PBS and centrifuged again under the same conditions before being resuspended in 500 µL sterile PBS. For in vivo imaging, mice were anesthetized with 2% isoflurane in oxygen and placed on a heated platform to maintain body temperature, and DiR-labeled NMM-EVs, DiR dye diluted in PBS (DiR-PBS), or PBS alone (50 µL per tibialis anterior muscle) were injected into the tibialis anterior muscle. Whole-body fluorescence images were acquired at 3 h, 1 d, 4 d, 7 d, and 14 d post-injection using an IVIS GS-5000 in vivo imaging system (Jingyi Technology Co., Ltd.) operated in fluorescence mode with identical acquisition settings for all groups.

### Histological staining

Paraffin-embedded tissue sections were prepared from the mid-belly of the tibialis anterior (TA) muscle and stained with hematoxylin and eosin (H&E), Masson’s trichrome, and Sirius Red. Transverse Sects. (8 µm thickness) were used for these histological analyses. H&E staining was used to evaluate overall muscle architecture and injury, based on the presence of inflammatory cell infiltration and myofiber damage. Regenerating myofibers were identified by centrally located nuclei. Masson’s trichrome staining was used to assess fibrosis, which was quantified as the percentage of fibrotic area relative to the total cross-sectional area (CSA). Sirius Red staining was used to visualize collagen fiber deposition associated with muscle injury and regeneration.

For Oil Red O staining, TA muscles were embedded in optimal cutting temperature (OCT) compound, snap-frozen, and cryosectioned at 8 µm thickness. Oil Red O staining was performed to detect adipocyte infiltration, and fat accumulation was assessed as the area containing Oil Red O–positive lipid droplets.

Morphometric analyses were performed using ImageJ software (NIH, Bethesda, MD, USA). For each staining method (H&E, Masson’s trichrome, Sirius Red, and Oil Red O), three sections per animal were analyzed. Sections were obtained from the mid-ventral region of the TA muscle, with a distance of 50 µm between adjacent sections to avoid repeatedly analyzing the same tissue region. For each section, at least five regions of interest (ROIs) were evaluated.

For H&E staining, ROIs were selected by two independent investigators blinded to group allocation using ImageJ. ROIs were defined as areas of muscle tissue showing evidence of damage, including inflammatory cell infiltration (densely packed small, hyperchromatic nuclei) and myofiber disruption (irregular fiber morphology, increased interstitial space, and presence of cellular debris). For Masson’s trichrome staining, fibrotic areas (blue-stained regions) were identified by color thresholding in ImageJ, and fibrosis was quantified as the percentage of blue-stained area relative to the total cross-sectional area. For Sirius Red staining, ROIs (200 µm × 200 µm) were manually selected within intermuscular regions exhibiting positive Sirius Red staining (red/orange birefringence under polarized light); five ROIs were randomly selected per section, avoiding large blood vessels and staining artifacts, and the Sirius Red–positive area fraction within each ROI was measured using ImageJ. For Oil Red O staining, ROIs were randomly selected within the muscle belly, and the fraction of Oil Red O–positive area within each ROI was measured using ImageJ.

### Grip strength

Grip strength was measured using a computer-controlled grip dynamometer (Jiangsu Saionsi Biotechnology Co., Ltd) equipped with a T-shaped metal bar connected to a force sensor. Hindlimb grip strength was assessed on days 1, 4, 7, and 14 after injury. Mice were gently held by the base of the tail and allowed to grasp the metal bar with their hind paws. To prevent interference from the forepaws, mice were first guided to grasp a metal mesh cylinder with their forepaws. Once the hind paws had firmly grasped the sensor bar, the mouse was steadily pulled backward by the tail until the grip was released. The peak force (in Newtons) was automatically recorded by the device. Each mouse underwent three consecutive grip strength measurements, and the mean value of the three trials was used for analysis.

### Immunofluorescence

#### Immunofluorescence staining of cultured cells

Immunofluorescence staining was performed to evaluate myogenic differentiation and cell proliferation in satellite cells (SCs) or primary satellite cells. Cells were stained with the following primary antibodies: anti-Pax7 (1:200, bs-5080R, BIOSS, China), anti-MyHC (1:150, M4276, Sigma, St. Louis, MO, USA), and anti-Ki67 (1:400, ab15580, Abcam, UK). Cells were first washed three times with PBS and fixed in 4% paraformaldehyde (PFA) for 15 min at room temperature. After additional PBS washes, cells were permeabilized with 0.25% Triton X-100 for 30 min and blocked in 5% goat serum for 1 h at room temperature. Primary antibodies were applied and incubated overnight at 4 °C. After washing with PBS, cells were incubated with Alexa Fluor–conjugated secondary antibodies (1:500; Thermo Fisher Scientific, USA) for 1 h at room temperature: Pax7 and Ki67 were detected using Alexa Fluor 488–conjugated goat anti-rabbit IgG (1:500, A56580, Thermo Fisher Scientific, USA), and MyHC was detected using Alexa Fluor 488–conjugated goat anti-mouse IgG (1:500, A28175, Thermo Fisher Scientific, USA). Nuclei were counterstained with Hoechst 33,342 (1:400, 10 µg/mL, Sigma). Immunofluorescence images were acquired using a confocal microscope (FV3000, Olympus, Tokyo, Japan). The fusion index was calculated as the number of nuclei within MyHC⁺ myotubes divided by the total number of nuclei, and the average number of nuclei per myotube was also determined.

#### Immunofluorescence staining of TA muscle cryosections

For immunofluorescence staining of tibialis anterior (TA) muscle, TA muscles were harvested and immediately frozen in isopentane cooled with liquid nitrogen, then embedded in optimal cutting temperature (OCT) compound. Cryosections (8–10 µm thick) were cut from the mid-belly of the muscle and processed for immunostaining. Sections were washed three times in PBS to remove OCT and then permeabilized with 0.25% Triton X-100 for 30 min at room temperature. Blocking was performed with 5% goat serum for 1 h at room temperature. The following primary antibodies were applied overnight at 4 °C: anti-laminin (1:200, ab11575, Abcam, UK), anti-eMyHC (1:200, F1.652, DSHB, USA), anti-collagen I (1:200, ab34710, Abcam, UK), anti-α-SMA (1:400, ab7817, Abcam, UK), and anti-Pax7 (1:200, bs-5080R, BIOSS, China). After washing with PBST (PBS + 0.1% Tween-20), sections were incubated with Alexa Fluor–conjugated secondary antibodies (1:500; Thermo Fisher Scientific, USA) for 1 h at room temperature: Pax7, α-SMA, and collagen I were detected using Alexa Fluor 488–conjugated goat anti-rabbit IgG (1:500, A56580, Thermo Fisher Scientific, USA), and eMyHC was detected using Alexa Fluor 555–conjugated goat anti-mouse IgG (1:500, A28180, Thermo Fisher Scientific, USA). Nuclei were counterstained with DAPI (10 µg/mL, Sigma). Imaging was performed with a confocal microscope (FV3000, Olympus), and image analysis was conducted using ImageJ software. At least five randomly selected injury sites were analyzed per section. Quantitative parameters, including the number of Pax7⁺ satellite cells, the number of eMyHC⁺ myotubes, collagen-positive area, and myofiber cross-sectional area (CSA), were normalized to the total tissue area.

### Myofiber isolation and culture

Single myofibers were isolated from the extensor digitorum longus (EDL) muscles of 6–8-week-old C57BL/6 mice as previously described (Tsuchiya and Ono 2022). Briefly, EDL muscles were enzymatically digested, and individual myofibers were released by gentle trituration and washed in Dulbecco’s modified Eagle’s medium (DMEM). Isolated myofibers were then cultured in DMEM supplemented with 20% fetal bovine serum (FBS) and 1% penicillin–streptomycin at 37 °C in a humidified atmosphere of 5% CO₂. After 42 or 72 h of culture, myofibers were fixed in 4% PFA in PBS for 10 min at room temperature and subsequently subjected to immunostaining as described in Sect. 4.10.

### CCK-8 assay

For the Cell Counting Kit-8 (CCK-8) assay, 1 × 10^4^ satellite cells (SCs) were seeded into 96-well plates and incubated with NMM-EVs for 24 h or 48 h. After incubation, 10 µL of CCK-8 solution was added to each well containing 100 µL of complete growth medium. Wells containing 100 µL of complete medium and 10 µL of CCK-8 solution but no cells served as blank controls. Cells were incubated in the dark at 37 °C in a humidified atmosphere of 5% CO₂ for 4 h, and the optical density (OD) at 450 nm was measured using a microplate reader.

### Serum creatine kinase (CK) assay

Serum creatine kinase (CK), a marker of muscle damage, was quantified using a commercial assay kit (Beyotime, Shanghai, China). Blood samples were collected via retro-orbital puncture and allowed to clot for 30 min at room temperature. After clotting, samples were centrifuged at 3000 rpm for 15 min at 4 °C to obtain serum. Serum CK levels were measured according to the manufacturer’s instructions, and absorbance at 570 nm was recorded using a microplate reader.

### Myogenic differentiation assay

Satellite cells (SCs) were seeded into six-well plates. Once cells reached approximately 80% confluence, the growth medium was replaced with differentiation medium consisting of high-glucose Dulbecco’s modified Eagle’s medium (DMEM) supplemented with 2% horse serum and 1% penicillin–streptomycin. Cells were maintained at 37 °C in a humidified atmosphere of 5% CO₂, and myotube formation was assessed by fluorescence microscopy after 6 days of differentiation.

### Wound healing assay

Satellite cells (SCs; 3 × 10^5^ cells) were plated in six-well plates and cultured until reaching approximately 70% confluence (about 24 h). Cells were then treated with extracellular vesicles and incubated at 37 °C for 4 h. A linear wound was created in the adherent monolayer using a sterile pipette tip, and the wells were gently washed with PBS to remove detached cells. Cells were subsequently incubated in fresh medium at 37 °C, and wound closure was monitored by imaging the scratched area at 0, 24, and 48 h.

### RNA extraction and real-time polymerase chain reaction (PCR)

Total RNA was extracted from the right tibialis anterior (TA) muscle using an RNA purification kit (R401-01, Vazyme Biotech, China) according to the manufacturer’s instructions. RNA concentration and purity were assessed using a NanoDrop 2000 spectrophotometer (Thermo Fisher Scientific, USA). Reverse transcription was performed using the PrimeScript™ RT Reagent Kit with gDNA Eraser (RR037A, Takara, Japan) to synthesize complementary DNA (cDNA) from 1 µg of total RNA. Quantitative real-time PCR (qPCR) for mRNA expression was carried out using a StepOnePlus™ Real-Time PCR System (Applied Biosystems, USA) in a 20 µL reaction containing 10 µL 2 × SYBR Green qPCR Master Mix (Vazyme Biotech), 1 µL cDNA template, 0.4 µL of each primer (10 µM), and 8.2 µL nuclease-free water. The thermal cycling conditions were as follows: 95 °C for 10 min, followed by 40 cycles of 95 °C for 15 s and 60 °C for 60 s. All primers were synthesized by Sangon Biotech (Shanghai, China), and the sequences are listed in Supplementary Table 1. β-Actin was used as the reference gene, and relative mRNA expression levels were calculated using the 2^ − ΔΔCt method.

For microRNA (miRNA) analysis, total miRNA was extracted from cells, muscle tissue, or NMM-EV preparations using the miRNeasy® Mini Kit (Qiagen, Germany) according to the manufacturer’s protocol. Reverse transcription of miRNAs was performed using the Mir-X™ miRNA First-Strand Synthesis Kit (Takara, Japan), and qPCR was conducted using the miScript® SYBR® Green PCR Kit (Qiagen, Germany). Each 20 µL reaction contained 10 µL SYBR Green Master Mix, 1 µL cDNA, 0.5 µL of each forward and reverse primer (10 µM), and 8 µL nuclease-free water. U6 small nuclear RNA (RNU6-1) served as the endogenous control for normalization. Relative expression levels of target miRNAs were calculated using the 2^ − ΔΔCt method. All reactions were performed in triplicate.

### Western blotting

Western blot analysis was performed on tibialis anterior (TA) muscle samples collected from mice at days 1, 4, 7, and 14 after intervention. Tissues were homogenized in ice-cold RIPA lysis buffer (Beyotime, Shanghai, China) containing protease inhibitors, and protein concentration was determined using a BCA Protein Assay Kit (Beyotime, Shanghai, China). Equal amounts of protein (20–40 µg) were separated by SDS-PAGE and transferred onto PVDF membranes (Millipore, USA). After blocking with 5% non-fat milk for 1 h at room temperature, the membranes were incubated overnight at 4 °C with the following primary antibodies: anti-PAX7 (bs-23668R), anti-MYOD (bs-1378R), anti-eMyHC (bs-70370R), anti-MYOG (bs-1299R), anti-MYHC (bs-9863R), anti-DESMIN (bs-1135R), anti-MF-20 (bs-10985R), anti-ACTIN (bs-0061R), and anti-TUBULIN (bs-4513R) (all from BIOSS, Beijing, China; 1:2000). After washing, membranes were incubated with HRP-conjugated goat anti-mouse or goat anti-rabbit secondary antibodies (BIOSS, 1:5000) for 1 h at room temperature. Protein bands were visualized using enhanced chemiluminescence (ECL) reagents (Thermo Fisher Scientific, USA) and captured using a ChemiDoc™ MP imaging system (Bio-Rad, USA). Band intensities were quantified with ImageJ software, and the relative expression levels of target proteins were normalized to TUBULIN.

### Statistical analysis

All data are presented as the mean ± standard error of the mean (SEM). Statistical analyses were performed using GraphPad Prism version 9.0 (GraphPad Software, San Diego, CA, USA). Comparisons between two groups were conducted using unpaired two-tailed Student’s t-test, while comparisons among more than two groups were assessed by one-way analysis of variance (ANOVA) followed by Tukey’s post hoc test, when applicable. A *P*-value of less than 0.05 was considered statistically significant. Statistical significance was denoted as follows: *P* < 0.05 (*), *P* < 0.01 (**), *P* ≤ 0.001 (***), and ns.: not significant (*P* ≥ 0.05).

## Supplementary Information

Supplementary Material 1. Figure S1. The number of delicate satellite cells in young mouse muscles is high and extracellular vesicles can activate the quiescent satellite cell pool. A, B Immunofluorescence staining of PAX7 (green) and DAPI (blue) in frozen sections of muscles from con groups and extracellular vesicles groups, old (18 months) groups and newborn (3 days) groups mice after extracellular vesicles treatment and untreated mice. Scale bar = 100 μm and 50μm, *n* = 6. C, D Western blot analysis of PAX7 expression in muscles from mice in each treatment group (*n* = 4, all compared with each group).

## Data Availability

The datasets generated and/or analyzed during the current study are available from the corresponding author on reasonable request.

## Abbreviations

SCs: Satellite cells
EVs: Extracellular vesicles
NMM-EVs: Neonatal mouse muscle–derived extracellular vesicles
TA: Tibialis anterior
FAPs: Fibroadipogenic progenitor cells
CSA: Cross-sectional area
miRNA: MicroRNA
