# Supplementary figures and images for: Efficacy of neonatal mouse muscle extracellular vesicles in skeletal muscle repair and regeneration
Source: Cell Regen. 2026 Jan 23;15:6. doi: 10.1186/s13619-025-00274-6 (PMC12827838; doi:10.1186/s13619-025-00274-6)

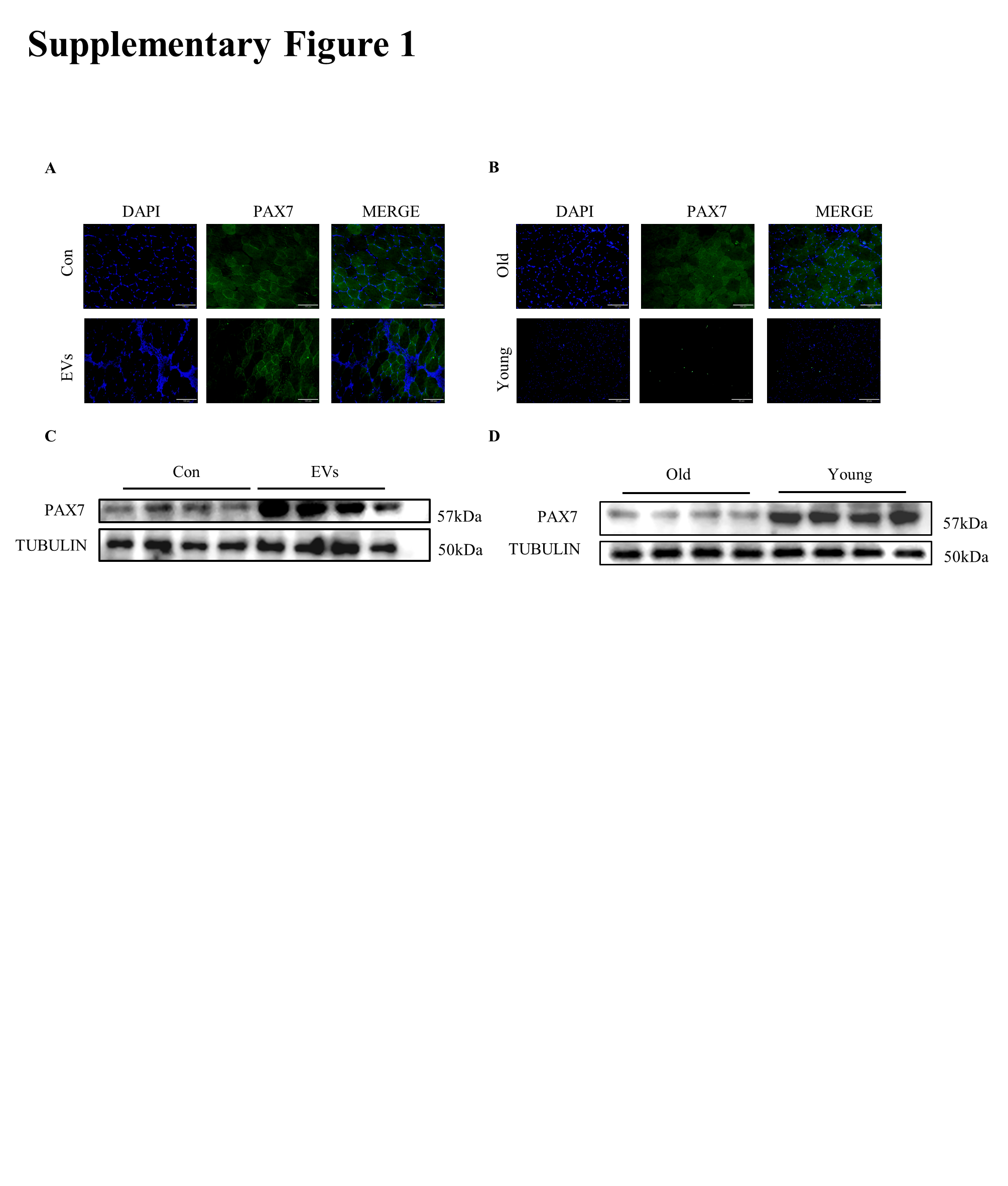

Supplement: Supplementary file 1 — Supplementary Material 1. Figure S1. The number of delicate satellite cells in young mouse muscles is high and extracellular vesicles can activate the quiescent satellite cell pool. A, B Immunofluorescence staining of PAX7 (green) and DAPI (blue) in frozen sections of muscles from con groups and extracellular vesicles groups, old (18 months) groups and newborn (3 days) groups mice after extracellular vesicles treatment and untreated mice. Scale bar = 100 μm and 50μm, n = 6. C, D Western blot analysis of PAX7 expression in muscles from mice in each treatment group (n = 4, all compared with each group). [file 13619_2025_274_MOESM1_ESM.tif]
